# Case Report: Primary intracranial lymphoma and meningioma manifesting as a composite tumor in a cat

**DOI:** 10.3389/fvets.2025.1619792

**Published:** 2025-08-07

**Authors:** Christina R. Vezza, Teresa L. Southard, Tanya LeRoith, Natalia J. Strandberg, Kayla M. Fowler, Richard L. Shinn, John H. Rossmeisl, Rell L. Parker

**Affiliations:** ^1^Department of Small Animal Clinical Sciences, Virginia-Maryland College of Veterinary Medicine, Virginia Polytechnic Institute and State University, Blacksburg, VA, United States; ^2^Department of Biomedical Sciences and Pathobiology, Virginia-Maryland College of Veterinary Medicine, Virginia Polytechnic Institute and State University, Blacksburg, VA, United States

**Keywords:** feline, composite tumor, case report, contiguous tumor, intracranial tumor

## Abstract

A 13-year-old, male neutered, Domestic Shorthair cat presented to the Virginia Tech Veterinary Teaching Hospital Neurology service for evaluation of episodes of altered mental status. On initial evaluation, the patient was noted to be alert and responsive, with a vestibular ataxia characterized by falling to the left and circling to the right. The neuroanatomic localization was consistent with multifocal intracranial disease affecting both brainstem and forebrain structures. A brain MRI revealed an extra-axial T2/FLAIR hyperintense, T1 isointense, strongly, homogenously, contrast-enhancing, plaque-like lesion affecting the meninges of the olfactory, frontal, parietal, and temporal areas of both cerebral hemispheres with extension into the falx cerebri and third ventricle. In the left temporal area, the plaque-like lesion was contiguous with a solitary, round, extra-axial, T2/FLAIR hypointense, T1 isointense, strongly and homogenously contrast-enhancing mass lesion. Bilateral caudal transtentorial and foramen magnum herniations were present. The cat’s neurologic status declined, and a left rostrotentorial craniectomy, regional durectomy, and temporal mass resection were performed. Both the meningeal plaque-like lesion and the temporal mass were sampled during surgery. The meningeal lesion presented two distinct neoplastic populations consistent with meningioma and large cell lymphoma, while the temporal mass cells were consistent with a meningothelial meningioma. The patient’s neurologic status improved postoperatively, and the cat was discharged on prednisolone therapy. The cat died 21 days after surgery, and a necropsy was performed. Gross examination revealed a plaque-like meningeal lesion involving the cerebrum. Histopathologically, the dura mater and subarachnoid space were infiltrated by sheets of CD79a-positive large neoplastic round cells, accompanied by numerous non-neoplastic CD3-positive T cells and IBA1-positive histiocytes, consistent with a T-cell-rich large B-cell lymphoma, and whorls of spindle-shaped cells, consistent with a meningioma. This is a rare case of an intracranial composite tumor of meningioma and primary central nervous system (CNS) T-cell-rich large B-cell lymphoma in a cat. Post-treatment survival in this cat was poor, similar to previously reported outcomes in cats with intracranial lymphoma.

## 1 Introduction

Intracranial neoplasia can be divided into primary brain tumors (PBTs) and secondary brain tumors (SBTs) (1). PBTs are neoplasms arising from the constitutive tissues of the brain parenchyma or surrounding meninges (2). The prevalence of PBTs in cats is approximately 2.2% based on necropsy data (3). PBTs make up approximately 70% of feline intracranial neoplasms (4). SBTs are neoplasms that metastasize to the brain from a distant site in the body or by direct extension from adjacent tissues (1). Metastasis can occur through hematogenous spread or through invasion by local extension from a site near the brain (1). The most common types of PBTs in cats are meningioma, followed by glial tumors (5).

Meningiomas account for 59% of all feline brain tumors and 85% of feline PBTs (4, 6). Affected cats are typically middle aged – older animals. Previous studies have shown that 17% of cats with meningiomas develop multiple, discrete mass lesions (4). Magnetic resonance imaging (MRI) features typically consist of broad-based extra-axial, T2W/FLAIR hyperintense, T1W isointense masses with strong, uniform contrast enhancement (6, 7). These tumors are most commonly slow-growing neoplasms that originate from arachnoid cap cells and grossly appear as well-demarcated, often lobulated, firm, or granular masses, although some can be cystic (6, 8). Histopathological subtypes of meningiomas differ based on the classification scheme used (6, 8–10). Co-immunolabeling for vimentin, CD34, and E-cadherin supports a diagnosis of canine and feline meningioma, though the degree of immunolabeling can be variable (11, 12). The prognosis for cats with palliative treatment for meningiomas is poor, with a median survival time (MST) of 18 days (4). Surgery is considered the treatment of choice for feline meningiomas, with an MST of 1,345 days (13). Radiation therapy may also be beneficial and is usually considered when surgery is not an option (13).

Lymphoma is the second most common intracranial tumor and the most common spinal cord neoplasm in cats (4, 5, 14, 15). Primary CNS lymphoma is described as uncommon, with only 35% of intracranial lymphomas classified as PBTs (4). In one study, 8 of 228 cats (3.5%) with intracranial tumors were diagnosed with primary CNS lymphoma (4). Affected cats have a median age of 10.5 years (4). MRI features of intracranial lymphoma vary in distribution (intra-axial, extra-axial, or both), but most lesions will enhance following contrast administration (16). Grossly, these tumors can manifest as white-tan, soft masses or regional swelling within the neuroparenchyma, leptomeninges (leptomeningeal lymphomatosis), or epidural space (14). On histopathology, lymphoma appears as atypical or monomorphic lymphocytes arranged in sheets (14, 15, 17). In a report on feline lymphoma of the nervous system, B-cell tumors accounted for 8/11 extra-axial masses, while leptomeningeal lymphomatosis mainly manifested with a T-cell immunophenotype (15). Histopathologic and immunohistochemical features of T-cell-rich large B-cell lymphoma include neoplastic large round cells that are immunoreactive to CD79a, CD20, and Pax5 and small lymphocytes immunoreactive to CD3 (14). The prognosis of intracranial lymphoma in cats treated with corticosteroids is poor, with a median survival time of 21 days (range 9–270 days) (4).

Rarely, two tumors can occur in the same anatomic location; this has been documented in both human and veterinary medicine (18–20). Synchronous tumors are two or more independent, macroscopically distinct neoplasms that arise simultaneously or within 6 months of each other. These tumors may be separate, or less commonly, contiguous (20–22). Among synchronous tumors, contiguous tumors are neoplasms that macroscopically appear as a single mass but histologically contain two or more discrete neoplastic populations. Contiguous tumors can be further classified into collision, composite (also called combined), or colonization tumors, depending on the histological relationship between the neoplastic populations (19, 22). Classification of contiguous tumors is based on the histologic borders, with collision tumors maintaining distinct borders between tumor phenotypes, composite tumors lacking distinct borders with intermingling of the neoplastic cellular populations, and colonization occurring when one tumor is completely encased within another (19, 23). The neoplastic populations in a composite tumor may also arise from the same oncogenic driver mutations (22). Contiguous tumors may arise when two adjacent neoplasms develop independently and then intermix with one another (19, 20). In general, contiguous tumors are rare. There are a few case reports of intracranial contiguous tumors in human and veterinary medicine (19, 20, 22, 24, 25). Several reported cases of intracranial contiguous or collision tumors consist of meningiomas and gliomas (21, 22). This case report describes the clinical, imaging, and neuropathologic features of an intracranial composite tumor consisting of meningioma and primary CNS T-cell-rich large B-cell lymphoma in a cat.

## 2 Case presentation

A 13-year-old, male neutered, Domestic Shorthair cat was repeatedly presented to an emergency hospital over a 1-week period for episodes of hiding and being found unresponsive by the owners. Bloodwork (CBC, chemistry, and fructosamine), urinalysis, abdominal radiographs, and abdominal ultrasound were performed and revealed a mild inflammatory leukogram (17.90; range 2.87–17.02 K/μL), thrombocytopenia (71; range 151–600 L/μL), hyperglycemia (244; range 71–159 mg/dL), hypokalemia (3.2; range 3.5–5.8 mmol/L), and hyperglobulinemia (5.9; range 2.8–5.1 g/dL). Cholecystitis, possible pyelonephritis, and nodules within the liver were noted on abdominal ultrasound. Treatment with marbofloxacin (4.3 mg/kg/day PO; Zeniquin, Zoetis Inc., Kalamazoo, MI, USA) was started, but there was no significant clinical improvement.

The cat was transferred to the Virginia Tech Veterinary Teaching Hospital Internal Medicine service for further investigation of cholecystitis. On initial examination, the cat was found to be bright, alert, and responsive. An intermittent 1/6 systolic parasternal heart murmur was auscultated. Neurologic examination revealed episodes of obtundation, as well as vestibular ataxia and circling to the right, with no other abnormalities noted. Bloodwork including a liver panel and chemistry panel revealed mild hypercalcemia (11.6; range 8.8–11 mg/dL), mild hyperproteinemia (8.7; range 6.6–8.5 g/dL), mildly decreased urea nitrogen (13; range 18–32 mg/dL), mildly increased GGT (1; normal is zero), and hypochloremia (108; range 116–123 mEq/L). Total T4 was within the reference interval at 21 nmol/L (range 16–37.7 nmol/L). The serum ammonia was considered normal (10 umol/L). The cat was tested and found to be positive for feline immunodeficiency virus (FIV) but negative for feline leukemia virus (FeLV). Thoracic radiographs revealed mild age-related changes, including mild bronchial wall thickening, and a focal area of mineralization within the abdomen, which was presumed to be a Bate’s body based on its appearance. A focused abdominal ultrasound showed a diffusely thickened gallbladder wall with a double-layered appearance. The liver had multiple poorly defined hyperechoic nodules, and the peritoneum had mild hyperechoic mesentery. A cholecystocentesis was performed without complications. Bile cytology showed crystalline structures but no bacteria or inflammatory cells, and culture was negative. A cardiology consultation and echocardiogram revealed an intermittent gallop rhythm and showed mild septal hypertrophy; no treatment was recommended. The systolic blood pressure was 150–160 mmHg using an oscillometric monitor, since the Doppler was not tolerated. The cat was treated with enrofloxacin (5 mg/kg IV q24; Baytril, Elanco US Inc., Greenfield, IN, USA), ampicillin/sulbactam (30 mg/kg IV q8; Unasyn; Pfizer, New York, New York, USA) buprenorphine (0.015 mg/kg IV q8; Buprenex, Indivior Inc., Raleigh, NC, USA) maropitant (1 mg/kg IV q24; Cerenia, Zoetis Inc., Kalamazoo, MI, USA), ondansetron (0.5 mg/kg IV q8; Pfizer, New York, New York, USA), and lactated Ringers solution at 11 mL/h IV (Abbott Laboratories, Abbott Park, IL, USA).

While hospitalized, the cat was noted to have a waxing and waning obtundation and was transferred to the neurology service. Upon examination at the time of transfer, the cat was bright, alert, and responsive, and a Grade II/VI parasternal heart murmur was noted. On gait evaluation, a vestibular ataxia with falling to the left was noted, and the cat displayed a tendency to circle (mid-size diameter) to the right. On cranial nerve evaluation, there was a decreased oculocephalic reflex bilaterally, worse when moving the head to the left. A right-sided Horner syndrome (ptosis, miosis, and enophthalmos) was identified. No other cranial nerve deficits were found. Postural reaction evaluation revealed decreased hopping in the right thoracic and pelvic limbs; the left thoracic limb and pelvic limbs had normal proprioception. Spinal segmental reflexes were normal. No other neurological examination abnormalities were identified.

**Figure 1:**
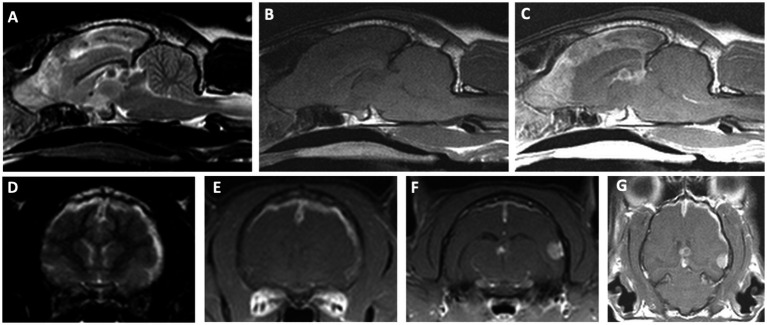
Brain magnetic resonance imaging in a cat with an intracranial composite tumor consisting of a meningioma and a primary CNS lymphoma. Top row: Sagittal T2W image **(A)** showing ill-defined heterogeneous T2W hyper- and hypointense signal along the falx cerebri extending into the olfactory bulbs. Note the round, well-demarcated T2W hypointense lesions within the dorsal aspect of the third ventricle and dorsal to the tectum. T1W pre-contrast sagittal **(B)** and T1W post-contrast sagittal **(C)** showing a diffuse and markedly contrast-enhancing lesion along the falx cerebri, extending into the olfactory bulbs, with peripheral contrast enhancement of the round lesions within the dorsal aspects of the third ventricle and tectum. Bottom row: For the transverse images, the left side of the image represents the right side of the patient. T2W transverse image **(D)** showing hyperintense and diffusely thickened meninges of the parietal and temporal regions, as well as midline shift. T1W post-contrast transverse showing markedly enhancing plaque-like lesion of the cerebral hemispheres extending along the falx cerebri and meninges of the parietal and temporal regions **(E)**, T1W post-contrast transverse at the level of the caudal thalamus showing an extra-axial, solitary, round, strongly contrast enhancing mass of the left temporal cortices at the level of the third ventricle **(F)**, T1W post-contrast dorsal view showing the extent of meningeal thickening, the solitary temporal mass, and the peripherally contrast enhancing lesion in the third ventricle **(G)**.

The neuroanatomic localization was consistent with multifocal intracranial disease involving both the forebrain and brainstem. The right-sided circling was attributed to a right forebrain lesion, although the presence of right-sided postural reaction deficits also suggested potential involvement of the left forebrain. Brainstem involvement, particularly the medulla, was suspected based on the presence of the diminished oculocephalic reflex and vestibular ataxia, and could also have contributed to the periodic obtundation. Given the other neurologic deficits present, an intracranial lesion in the brainstem was also suspected to be causing the Horner syndrome. Bilateral or diffuse forebrain disease was also considered as a possible localization for the obtundation. The cat was anesthetized, and a brain MRI was obtained (Phillips Intera 1.5 T MRI Scanner, Cleveland, OH), which revealed multiple intracranial lesions. There was a plaque-like mass of the cerebral pachymeninges, approximately 1 mm thick, involving predominantly the dorsal and lateral, and medial aspects of the olfactory bulbs, frontal, parietal, and temporal lobes of the right and left cerebral hemispheres, with extension along the falx cerebri into the third ventricle (Figure 1). The pachymeningeal lesion was extended more laterally and ventrally in the right cerebral hemisphere. This tissue was extra-axial, T2W/FLAIR heterogeneously hyperintense and hypointense, T1W isointense, strongly and homogeneously contrast-enhancing. The pachymeningeal lesion involved 44% of the total cerebral meningeal area. This was calculated using a volumetric surface area mask of the abnormal, contrast-enhancing regions of the cerebral meninges divided by the volumetric surface area mask of the entire cerebral meninges. This was performed using the area function of Osirix MD (v11.0.4, Pixmeo, Switzerland). Discrete round T2W hypointense and peripherally contrast-enhancing lesions associated with signal voids on susceptibility-weighted imaging were noted in the dorsal aspect of the third ventricle and dorsal to the tectum. Contiguous with the plaque-like meningeal lesion, there was a solitary, round extra-axial mass in the superficial aspect of the left temporal area that measured 6.6 mm x 5.8 mm x 5.4 mm (Figures 1F, G). This mass was T2W/FLAIR hypointense, T1W isointense, intensely and homogeneously contrast-enhancing. Calvarial hyperostosis was present overlying the left temporal mass. There was mild deviation of the falx cerebri to the right. Bilateral caudal transtentorial herniations and mild foramen magnum herniation were also present. Syringohydromyelia was also noted in the cranial cervical spinal cord. The main differentials were neoplasia (including meningioma, lymphoma, histiocytic sarcoma, and granular cell tumor), with infectious and autoimmune meningoencephalitis and hypertrophic pachymeningitis considered less likely. The cat recovered uneventfully from general anesthesia following the brain MRI.

The next day, at the time of planned discharge, the cat became obtunded to stuporous and was laterally recumbent with mydriatic pupils and absent pupillary light reflexes bilaterally. Postural reactions were absent in all four limbs. A limited examination was completed at this time due to the critical nature of the cat’s condition. The heart rate was 200 BPM, and the Doppler blood pressure was 200 mmHg. Increased intracranial pressure was suspected. The cat’s mentation temporarily improved following administration of mannitol (1 g/kg IV; Abbot Laboratories) and hypertonic saline (3 mL/kg IV; Abbott Laboratories). The owners consented to an emergency craniectomy with the goals of reducing presumed intracranial hypertension via debulking of the lesions and obtaining a histopathologic diagnosis for the intracranial masses.

**Figure 2:**
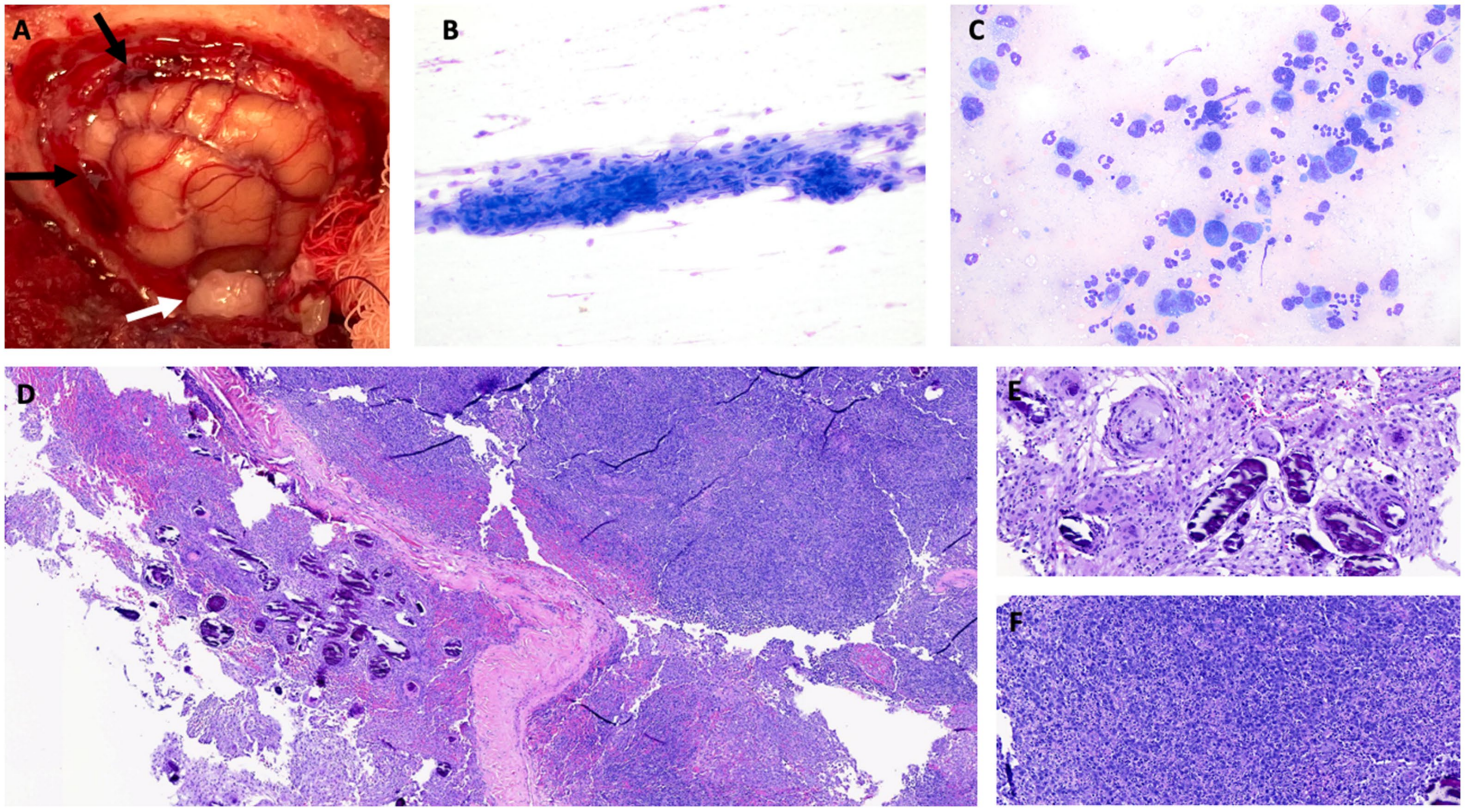
**(A)** Intraoperative image after craniectomy showing the left cerebral hemisphere after durectomy and hemorrhagic meninges dorsally and rostrally, marked by black arrows. The solitary mass of the left temporal area is marked by a white arrow. **(B)** Cytology of the temporal mass. Aggregated fusiform cells with oval nuclei and minimal anisocytosis and anisokaryosis, cytologically most consistent with meningioma. Modified Wright stain, 200x magnification. **(C)** Cytology of meninges. The cells consist of large atypical round cells as well as inflammatory cells (predominantly non-degenerate neutrophils). The large atypical round cells have a moderate to high nuclear-to-cytoplasmic ratio. The nuclei are irregular to highly lobulated, and the cytoplasm is medium to deep blue. The morphology, together with the atypical nuclei, is most consistent with neoplastic large lymphocytes. Modified Wright stain, 500x magnification. Histopathologic image of hematoxylin and eosin (H&E)-stained tissue sample obtained by biopsy showing low power **(D)** view of meningioma with psammoma bodies, with higher power view of psammoma bodies provided in **(E)** (H&E, 100x). **(F)** High power view of densely cellular meningeal infiltrate of neoplastic lymphocytes (H&E, 100x).

A left rostrotentorial craniectomy was performed, which extended from the bregma and the sagittal suture to the level of the zygomatic arch (Figure 2A) (26). Throughout the surgical field, the meninges were thickened, opaque, yellow-tan discolored, and contained hemorrhagic foci noted dorsally and rostrally. A regional durectomy was performed, which revealed a tan subdural exudate present diffusely on the surface of the exposed brain. A pale-yellow, round, 1 cm extra-axial mass at the level of the zygomatic arch caudal to the ectosylvian gyrus, compressing the brain, was also observed and removed en bloc. Samples of meninges and temporal mass were obtained and submitted for cytology, culture, and histopathologic analysis. A porcine small intestinal submucosa graft (SIS; Cook Biotech, West Lafayette IN, USA) was placed over the dural defect and a titanium mesh (Kinamed NeuroPro, Camarillo, CA, USA) cranioplasty performed, held in place with three 1.5 mm x7 mm titanium screws (Kinamed NeuroPro). The cat recovered uneventfully from general anesthesia. The following day, the cat’s mentation was normal, and the only remaining neurologic abnormalities were a decreased oculocephalic reflex (improved compared to pre-operative exam) and incomplete pupillary light reflexes bilaterally. The cat was discharged 3 days after surgery with a static neurologic examination when compared to the immediate postoperative examination and was treated with prednisolone (1 mg/kg/day PO; Abbott) and gabapentin (10 mg/kg q8 PO; Actavis, Troy-Hills, NJ, USA).

Intraoperative cytology of the meninges revealed a mixed population of inflammatory cells and large mononuclear cells (Figure 2C). The large mononuclear cells had a high nuclear-to-cytoplasmic ratio and were most consistent with atypical lymphocytes. The nuclei were irregular to heavily lobulated and had an open chromatin pattern. Rare binucleation and mitotic figures were noted. Rare fusiform cells were noted in the meningeal sample. Cytologic diagnosis of the meningeal sample was suspected of large cell lymphoma. Cytology of the temporal mass showed fusiform nucleated cells in aggregates with some capillary elements. The cells had light blue cytoplasm and indistinct borders with mild anisocytosis and anisokaryosis, which was interpreted as most consistent with a meningioma (Figure 2B). Aerobic and anaerobic cultures of all surgically obtained tissue samples were negative.

Samples of the hyperostotic skull, thickened meninges, and the entire left temporal mass were submitted for histopathologic analysis. The skull was decalcified and considered normal on histopathologic examination. On histopathology of the meninges, there was a discrete, densely cellular mass of spindle cells, often forming whorls around areas of mineralization (psammoma bodies) (Figures 2D,E). The meninges were infiltrated with neoplastic round cells arranged in sheets (Figure 2F). These cells had scant eosinophilic cytoplasm and indistinct borders. The nuclei were round, variable in size (up to twice the diameter of an erythrocyte), and had finely stippled chromatin. Anisocytosis and anisokaryosis were moderate, and the mitotic count for the round cells was 51 in 2.37 mm^2^ (10 40x fields). There was extensive individual cell necrosis with infiltration by neutrophils. Features were consistent with a diagnosis of a meningioma with a prevalent psammomatous pattern and a round cell tumor, likely a large cell lymphoma. The temporal mass was composed of tightly packed spindle cells arranged in sheets. The cells had abundant eosinophilic granular cytoplasm, and the mitotic count was 3 in 2.37 mm^2^ (10 40x fields). The temporal mass was diagnosed as a meningothelial meningioma.

The owners consulted with an oncologist but elected not to pursue any treatment beyond the use of corticosteroids. Gabapentin was discontinued due to excessive sedation. The cat was re-evaluated by the VTH Neurology service 2 weeks after discharge. He had been doing well at home, but 4 days prior to the visit, he was noted to be lethargic with a decreased appetite and difficulty chewing; dysphagia was suspected. The cat’s weight decreased from 5.35 kg at the initial visit to 4.4 kg. The surgical incision had healed. During the neurologic examination, the cat was mildly obtunded and ambulatory with no apparent ataxia, paresis, or circling. There was a decreased oculocephalic reflex and mild anisocoria with slight miosis in the right eye. The neuroanatomic localization was either diffuse forebrain or brainstem. However, given the continued presence of Horner syndrome, forebrain involvement was considered less likely. The owners elected to continue palliative care, and the cat died at home 21 days after surgery. The owners brought the patient to Virginia Tech Veterinary Teaching Hospital for a necropsy.

**Figure 3:**
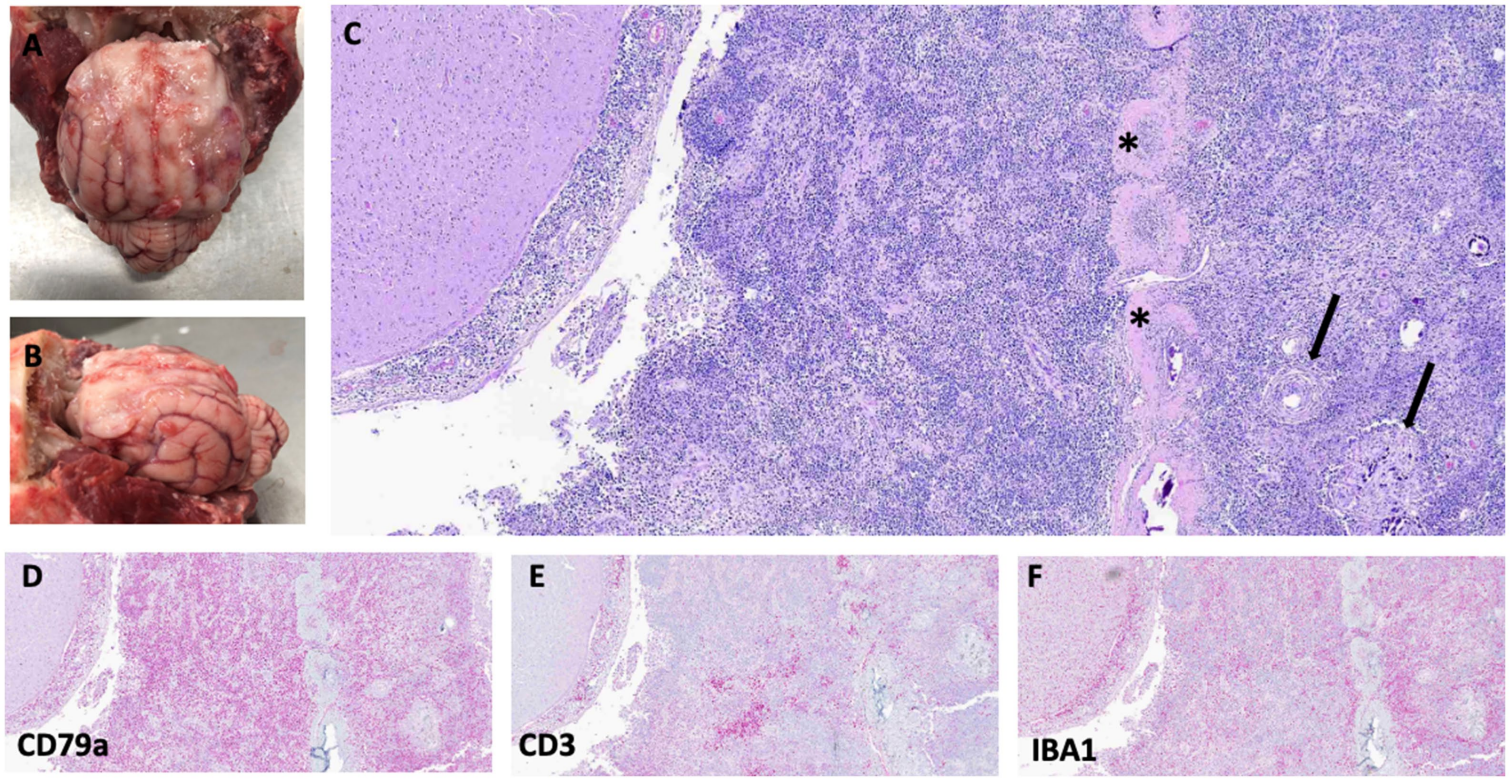
Gross images showing plaque-like thickening and irregularity of meninges in the frontal region of the brain and extending into the left and right sides of the cerebral hemispheres **(A,B)**. Photomicrographs of hematoxylin and eosin (H&E)-stained slides of the meninges with regions of meningioma cells (arrows) mixed with neoplastic round cells surrounding the falx cerebri (asterisks) **(C)**. Bottom row: All stained with H&E, 40x. **(D)** CD79a (B-cell marker), **(E)** CD3 (T-cell marker), and **(F)** IBA1 (macrophage marker) immunostaining showing strong cytoplasmic immunoreactivity for CD79a, with fewer cells exhibiting immunoreactivity with CD3 and IBA1. For immunohistochemistry details, see Supplementary Table 1.

During necropsy, significant findings were limited to the brain. Grossly, the titanium mesh cranioplasty was in place. Removal of the titanium mesh revealed a firm, tan, up to 2-mm thick, plaque-like mass expanding the meninges along the margins of the prior durectomy, which was adhered to the frontal region of the cerebrum. The cerebellum was coned at the foramen magnum (Figures 3A,B). No evidence of extracranial neoplasia was found on gross or microscopic examination.

Histologically, the pachymeninges and leptomeninges were expanded by sheets of large (up to twice the diameter of an erythrocyte) atypical round cells with scant granular cytoplasm and round to lobulated nuclei, moderate anisocytosis and anisokaryosis, and 45 mitotic figures in 2.37 mm^2^ (10 40x fields) (Figure 3C). Mixed among these neoplastic cells was a population of smaller, non-neoplastic lymphocytes. Immunohistochemical analysis of the tumor was performed using antibodies and techniques as described in Supplementary Table 1 and the Supplementary methods. The large, atypical round cells were positive for CD79A (a B-cell marker) on immunohistochemistry, while the smaller lymphocytes were positive for CD3 (T-cells), consistent with a T-cell-rich B-cell lymphoma (Figures 3D,E). Additionally, a population of IBA1-positive macrophages infiltrated the tumor (Figure 3F). Adjacent to and within the round cell neoplasm were clusters and whorls of spindle cells, sometimes surrounding psammoma bodies, with mild anisocytosis and anisokaryosis, and no mitotic figures in an area of 2.37 mm^2^ (10 40x fields). This neoplasm was diagnosed as a meningioma (Figure 3C).

## 3 Discussion

In this study, we describe a meningioma and primary CNS lymphoma composite tumor in a cat. Although meningiomas are common, primary CNS lymphoma is uncommon in cats, and a contiguous, composite tumor consisting of these two PBTs has not previously been reported in detail in cats. Approximately 14% of cats with a meningioma also have another tumor, such as lymphoma or a pituitary tumor, which we interpret as being synchronous (4). In reported cases of synchronous meningioma and lymphoma in cats, the lymphoma was typically secondary (i.e., a secondary brain tumor, or SBT) (4). Reports of contiguous tumors in veterinary medicine are scarce, with one described case involving an intracranial collision tumor composed of a meningioma and a glioma in a dog (21).

Contiguous tumors can consist of either benign or malignant lesions, and the boundaries between the two tumor types may be distinct or the tumors may permeate into one another. These are further subdivided by the degree of histopathologic overlap (19, 23). In the current case, the two cell populations were found to be intertwined in both the biopsy and necropsy samples, so this case is considered to represent a composite tumor variant of a contiguous tumor (19). However, the nomenclature for contiguous tumors is not well defined.

Based upon the necropsy findings, the pattern of growth for the lymphoma in this report was most consistent with leptomeningeal lymphomatosis, although conclusive evidence of leptomeningeal disease was not apparent on the MRI. This was the pattern seen in 4 of 22 recently reported feline primary intracranial lymphomas (15). According to immunophenotyping, the tumor in this cat was a T-cell-rich B-cell lymphoma; B-cell lymphoma was reported in 12 of 22 of the recent cases as well (15).

This cat was FIV positive, which increases the risk of developing lymphoma by approximately fivefold (27, 28). Cats with FIV frequently develop B-cell lymphoma but are also predisposed to other neoplasms (27). Therefore, this cat had an increased risk of developing lymphoma, which may have increased the likelihood of developing contiguous tumors.

In the present case, although the meningioma contained areas with a high density of psammoma bodies, the predominant histological pattern present was considered meningothelial. A rare but important differential diagnostic consideration for the intracranial lesion noted in this cat could also include lymphoplasmacyte-rich meningioma. In humans, these are WHO grade I meningiomas that may manifest with an en plaque phenotype similar to the MRI appearance of the cat described here and histologically demonstrate dense, lymphoplasmacytic infiltrates in a meningothelial background (29). This type of meningioma is often softer than typical meningiomas on gross examination (29). This was considered an unlikely diagnosis in this case as the CD79a immunoreactive round cells had a very high mitotic count and showed features of cellular atypia that would not be expected in a lymphoplasmacyte-rich meningioma. The absence of plasma cells and macrophages within the meningeal infiltrate also made lymphoplasmacyte-rich meningioma less likely (29).

The cat described here initially presented with subtle and intermittent clinical signs, which were originally attributed to cholecystitis. This is a frequent scenario in cats with brain tumors, where initial clinical presentations are often non-specific (4, 13). The most common clinical signs in cats with brain tumors are altered mental status, circling, seizures, and lethargy with decreased appetite (4, 13). In this case, the cat experienced acute progression of clinical signs while hospitalized, culminating in catastrophic deterioration following anesthesia, which was attributed to intracranial hypertension or brain herniations. While cats with brain tumors, particularly meningiomas, often show neuroimaging evidence of brain herniations and intracranial hypertension, many do not exhibit overt clinical manifestations of these changes (30, 31).

While the morphology and appearances of the MRI lesions present in this cat demonstrated many overlapping features compatible with those previously described for lymphoma and meningioma, as well as with some meningoencephalitides, there were also some unusual characteristics (4–6, 14) Extensive and markedly enhancing meningeal lesions can be seen with lymphoma or en plaque meningiomas, although the hypointense T2W/FLAIR signal noted in the temporal lobe meningioma of this case would be atypical for lymphoma (14, 32). Furthermore, this case demonstrated focal calvarial hyperostosis, which, while common in cats with meningioma, is not a reported feature of lymphoma (14, 32).

This cat had a positive but transient clinical response to surgical treatment, possibly due to debulking of the lesion, reducing intracranial pressure, or both, and surgery allowed for definitive histopathologic diagnosis (14). While surgery is the treatment of choice for feline meningiomas, its potential role as a therapeutic modality for management of CNS lymphoma is currently unknown (4, 13, 14). Given the diffuse and infiltrative nature of CNS lymphoma, multiagent chemotherapeutic protocols or radiotherapy are usually recommended to extend survival, but there is limited evidence supporting the efficacy of these approaches in cats with histologically confirmed tumors, and the prognosis associated with CNS lymphoma is poor (4, 14).

In summary, this is the first detailed description of an intracranial composite tumor of meningioma and primary CNS T-cell-rich large B-cell lymphoma in a cat. While synchronous brain tumors are not unusual in cats, a contiguous tumor should be considered as a differential diagnosis in cases that have lesions with MRI phenotypes that are unusual or overlap with multiple tumor types. Survival after surgery and with corticosteroid monotherapy was poor in this case, which is similar to previous reports on MST for intracranial lymphoma in cats receiving similar treatment (4).

## Data Availability

The original contributions presented in the study are included in the article/Supplementary material, further inquiries can be directed to the corresponding author.
