# Supplementary material for: Case Report: Primary intracranial lymphoma and meningioma manifesting as a composite tumor in a cat
Source: Front Vet Sci. 2025 Aug 7;12:1619792. doi: 10.3389/fvets.2025.1619792 (PMC12367500; doi:10.3389/fvets.2025.1619792)
Supplement: Supplementary file 1 [file Supplementary_file_1.docx]

Supplementary Methods:

Formalin-fixed, paraffin-embedded tissue sections were processed at the Virginia Tech Animal Laboratory Services lab in Blacksburg, VA, using standard protocols and an automated IHC stainer (Bond-Max automated IHC staining system; Leica). Briefly, serial 4-µm tissue sections mounted on charged slides were deparaffinized (AR9222, Bond dewax solution; Leica), and after heat epitope retrieval (AR9640, Bond epitope retrieval solution 2; Leica), the slides were incubated with the respective primary antibody for 15 minutes followed by a polymeric horseradish peroxidase (DS9800; Bond polymer refine detection; Leica) linker antibody conjugate detection system, and hematoxylin counterstain (DS9390; Leica) for 5 minutes. For T-cell detection, the ready-to-use mouse monoclonal IgG2a anti-human T-cell antigen receptor complex (PA0553, Ready-to-use primary antibody CD3, clone LN10; Leica) was used. For B-cell detection, ready-to-use mouse monoclonal IgG1 anti-human CD79a (PA0599, clone JCB117; Leica) was used. After a 5-minute peroxide block (DS9800; Leica), macrophages–dendritic cells (IBA1) were detected by using a rabbit polyclonal antibody raised against a conserved human, rat, and mouse synthetic peptide of the IBA1 C-terminal sequence (019-19741; Fujifilm Wako) diluted 1:3,000. Normal canine lymph node served as a positive antibody and method control for T and B cells, and for macrophage–dendritic cells; omission of the primary antibody on serial sections served as a negative method control. Positive immunolabeling was identified as brown staining with diaminobenzidine (DAB).
