# Supplementary material for: Case Report: Primary intracranial lymphoma and meningioma manifesting as a composite tumor in a cat
Source: Front Vet Sci. 2025 Aug 7;12:1619792. doi: 10.3389/fvets.2025.1619792 (PMC12367500; doi:10.3389/fvets.2025.1619792)
Supplement: Supplementary file 2 [file Table_1.docx]

Supplementary Table 1: Antibodies used for immunohistochemistry.

|  | Manufacturer | Source | Clonality | Retrieval | Dilution |
| --- | --- | --- | --- | --- | --- |
| CD79a | Santa Cruz | Mouse | Monoclonal | CC1 | 1:50 |
| CD3 | Dako | Rabbit | Polyclonal | CC1 | 1:100 |
| IBA1 | Fuji Film | Rabbit | Monoclonal | CC1 | 1:1000 |
